# Differentiating Zeranol Implant Abuse and *Fusarium* spp. Toxin-Contaminated Corn Intake by Detection and Quantification of Resorcylic Acid Lactones in Bovine Urine

**DOI:** 10.3390/toxins17070347

**Published:** 2025-07-11

**Authors:** Rafael Silva Gomes, Vanessa Gonçalves dos Santos, Carlos Juliano da Silva, Amanda Martinez Nagato Simões, Eliene Alves dos Santos, Mary Ane Gonçalves Lana, Kelly Moura Keller, Marco Blokland, Ane Arrizabalaga-Larrañaga, Rafael Romero Nicolino, Marcelo Resende de Souza, Tadeu Chaves de Figueiredo, Saskia Sterk, Silvana de Vasconcelos Cançado

**Affiliations:** 1 School of Veterinary, Federal University of Minas Gerais State (UFMG), Belo Horizonte 31270-901, Minas Gerais, Brazil; rafaelgomesvet@yahoo.com.br (R.S.G.); kelly.medvet@gmail.com (K.M.K.); rafael.nicolino@gmail.com (R.R.N.); marceloresende51@gmail.com (M.R.d.S.); tadeucfigueiredo@gmail.com (T.C.d.F.); 2 Federal Laboratory of Animal and Plant Health and Inspection in São Paulo (LFDA-SP), Ministry of Agriculture (MAPA), Campinas 13100-105, São Paulo, Brazil; vanessa.dsantos@agro.gov.br (V.G.d.S.); carlos.juliano@agro.gov.br (C.J.d.S.); amanda.simoes@agro.gov.br (A.M.N.S.); 3 Federal Laboratory of Animal and Plant Health and Inspection in Minas Gerais (LFDA-MG), Ministry of Agriculture (MAPA), Pedro Leopoldo 33250-220, Minas Gerais, Brazil; salveseliene@hotmail.com (E.A.d.S.); mary.lana@agro.gov.br (M.A.G.L.); 4 Wageningen Food Safety and Research, European Union Reference Laboratory for Growth Promotors, Wageningen University & Research, 6700 HB Wageningen, The Netherlands; marco.blokland@wur.nl (M.B.); ane.arrizabalagalarranaga@wur.nl (A.A.-L.); saskia.sterk@wur.nl (S.S.)

**Keywords:** LC-MS/MS, food safety, mycotoxins, zeranol, growth promoter

## Abstract

Resorcylic acid lactones (RALs) are fungal metabolites with known biological activity. Zeranol, a synthetic RAL, has been used as an estrogenic growth promoter in cattle; however, its use is prohibited in several countries. Zearalenone, a mycotoxin produced by *Fusarium* spp., is commonly found in contaminated animal feed and can be metabolized into other RALs, which are subsequently excreted in urine. To differentiate between natural contamination from feed and the illegal administration of zeranol, the European Union Reference Laboratory for Growth Promoters (EURL) developed a mathematical equation. This study aims to evaluate the detection and quantification of RALs in bovine urine from animals fed zearalenone-contaminated diets, implanted with zeranol, or subjected to both conditions. RALs were detected and quantified in the urine of cattle consuming contaminated corn, while zeranol and taleranol were identified in the urine of implanted animals. The EURL equation proved to be a valuable tool for determining the origin of RALs in bovine urine and holds significant potential for monitoring and enforcing regulations regarding the illegal use of zeranol.

## 1. Introduction

α-Zearalanol (α-ZAL, commonly known as zeranol) is a semi-synthetic compound derived from the mycotoxin zearalenone (ZEN). It functions as a non-steroidal estrogenic growth promoter, commonly used in beef cattle to enhance live weight gain, improve feed conversion efficiency, and reduce carcass fat content [1,2,3]. Zeranol has been legalized in some countries, such as the United States, Canada, Australia, New Zealand and some countries in Asia and Africa [4,5]. However, due to concerns over its potential risks to human health, the use of zeranol has been banned in the European Union (EU) and in several other countries, including Brazil. Consequently, the monitoring of zeranol residues in food-producing animals is mandatory to ensure food safety and regulatory compliance [6,7,8,9].

When administered via subcutaneous implants, zeranol is gradually absorbed into the bloodstream. The highest concentrations of this anabolic steroid and its isomer, β-zearalanol (β-ZAL, taleranol), are typically detected at the implantation site, as well as in bile, feces, and urine [10]. Among these, urine is considered the matrix of choice for residue monitoring.

ZEN, classified as a non-steroidal resorcylic acid lactone (RAL), is a mycotoxin produced by *Fusarium* species, including *F. graminearum*, *F. culmorum*, *F. equiseti*, and *F. cerealis*, which commonly contaminate pastures and cereal grains [11,12]. Following ingestion by ruminants, ZEN undergoes metabolic transformation mediated sequentially by ruminal protozoa, enterocytes, and hepatocytes at successive stages of mycotoxin metabolism. Initially, ZEN is reduced by 3α- and 3β-hydroxysteroid dehydrogenases to α-zearalenol (α-ZEL) and β-zearalenol (β-ZEL), respectively. Subsequently, α-ZEL can be further reduced to zeranol, while β-ZEL is metabolized into taleranol [13,14]. Thus, zeranol and taleranol can occur naturally in the urine and bile of bovines after the metabolism of ZEN, and therefore, the presence of these substances in the urine of an animal may be insufficient proof that abuse has occurred. The former substances, α-ZEL, β-ZEL and ZEN are called *Fusarium* spp. toxins [15]. The principal biological effect of ZEN and its metabolites—particularly α-ZEL—is their potent estrogenic activity. These compounds structurally mimic the endogenous steroid hormone 17β-estradiol and competitively bind to estrogen receptors, leading to endocrine disruption and physiological effects on the reproductive system [16,17].

Control laboratories in the European Union (EU) conducted an epidemiological study aimed at developing a reliable method to differentiate between exogenous administration and the natural occurrence of RALs. A total of 8008 urine samples were screened using a time-resolved fluoroimmunoassay (TR-FIA) for the presence of zeranol, and 461 positive samples were analyzed using confirmatory mass spectrometry methods. Among them, 174 samples were classified as ‘equivocal’ due to the simultaneous detection of zeranol (or taleranol) and *Fusarium* spp. toxins. The concentrations of these compounds in the equivocal samples were used to develop a linear regression model with a 99% prediction interval. This model was designed to discriminate between RALs originating from the natural contamination of feed by *Fusarium* toxins and those resulting from the illegal use of zeranol implants. As a result, the concentration of the analyzed compounds in four samples was outside the expected range, and the presence of zeranol in these samples was considered to be due to the illegal use of the synthetic compound [18].

However, the samples used in that study were randomly collected from four countries within the European Union. To the best of our knowledge, no studies have validated whether the proposed equation can be extrapolated to animals outside Europe, where variations in the presence of *Fusarium* spp. toxins, as well as differences in animal metabolism, may arise due to climatic conditions or genetic and physiological diversity among animal populations. Considering these aspects, the objectives of this study were to determine the behavior of ZEN metabolites (feed contaminated with *Fusarium* spp. toxins) and/or zeranol (use of implant), over time, in bovine urine provided by a controlled animal experiment and also to apply the equation recommended by the European Union Reference Laboratory for Growth Promoters (EURL) to discriminate between natural contamination of the diet and the illegal use of growth promoter.

## 2. Results and Discussion

### 2.1. Detection and Quantification of RALs

Residues of the *Fusarium* spp. toxins (ZEN, α-ZEL, β-ZEL), zeranol and taleranol were not detected in any of the samples on day zero (control).

**Figure 1:**
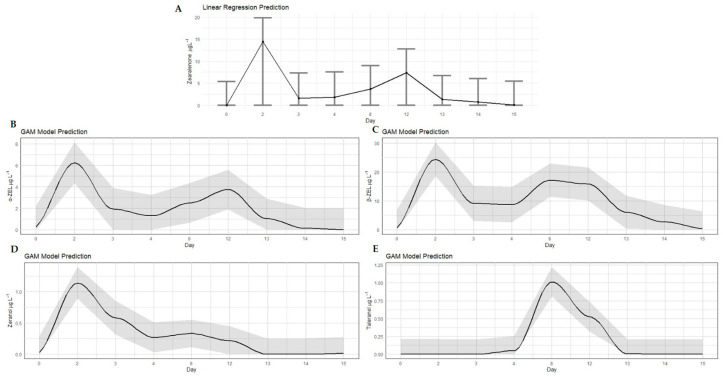
Concentrations of zearalenone (**A**), α-ZEL (**B**), β-ZEL (**C**), zeranol (**D**) and taleranol (**E**) residues in the urine of bovines fed ZEN-contaminated corn at a concentration of 2100 ± 200 µg per day for 12 days (treatment A). For ZEN (**A**), a linear regression model was used. The dark gray area surrounding the black line represents the confidence interval.

All RALs were detected and quantified in the urine of animals in treatment A, which received contaminated corn for 12 days (Figure 1; Table A1). Peaks with the highest concentrations of ZEN (14.45 µg L^−1^), α-ZEL (6.24 µg L^−1^) and β-ZEL (24.37 µg L^−1^) were observed on the second experimental day (*p* < 0.05), which demonstrates the rapid metabolism and excretion of ZEN by the animal organism (Table A1). On the third experimental day, the levels of *Fusarium* spp. toxins decreased; however, they increased again on the eighth and twelfth days of experimentation, reaching levels of ZEN, α-ZEL and β-ZEL of 3.63, 2.49 and 17.14 µg L^−1^ and 7.40, 3.74 and 15.80 µg L^−1^, respectively. The β-ZEL and ZEN concentrations in the urine were higher compared to that of the taleranol; similar results were found by Falkauskas et al. [19].

Zeranol and taleranol were also detected but at substantially lower concentrations than the primary *Fusarium* metabolites. Zeranol reached its peak on day two (1.08 µg L^−1^) following the initial intake of contaminated corn, while taleranol peaked on day eight (1.01 µg L^−1^) (*p* < 0.05). This pattern suggests that the conversion of α-ZEL to zeranol occurs more rapidly than the conversion of β-ZEL to taleranol. Taleranol was only quantified on day eight (*p* < 0.05), and low amounts of this metabolite were observed on day twelve. After 15 days, the presence of α-ZEL was no longer detected, while β-ZEL and ZEN were present as traces in only a few samples. One day after the removal of the contaminated corn (day 13), the presence of zeranol and taleranol was no longer detected in the urine of the bovines. During the entire experimental period, it was observed that the numeric values of α-ZEL were lower than the values of β-ZEL. Despite the lack of information on toxic kinetics in cattle, it is known that bovines are more resistant to the adverse effects of ZEN than other species of production animals as they metabolize ZEN mainly into β-ZEL and less into α-ZEL [13,17].

To the best of our knowledge, limited data are available in the literature regarding the detection and quantification of RALs in the urine of animals fed with *Fusarium* spp. toxins, and no studies have specifically addressed the combined assessment of dietary exposure and the use of zeranol implants. Kleinova et al. [20] fed bovines with oat contaminated with 2740 µg/day of ZEN for 84 consecutive days. They analyzed the urine of the animals and found, predominantly, β-ZEL (20 to 65 µg L^−1^) followed by ZEN (5 to 8 µg L^−1^) and α-ZEL (3 to 5 µg L^−1^). Zeranol (2 to 3 µg L^−1^) and taleranol (2 to 3 µg L^−1^) were also quantified.

Takagi et al. [21] observed that urine concentrations of ZEN and its metabolites did not change in samples collected from cattle beyond 2 h after being fed, irrespective of the ZEN contamination levels.

Salvat et al. [10] fed bovines with 1000 µg of ZEN per day from the first to the fifth day, and then with 2000 µg of ZEN per day from the sixth to the sixteenth day and quantified higher concentrations of β-ZEL (36.30 µg L^−1^), followed by ZEN (15.28 µg L^−1^) and α-ZEL (5.39 µg L^−1^), while zeranol and taleranol were detected at concentrations of 2.41 and 0.19 µg L^−1^, respectively, in the urine.

**Figure 2:**
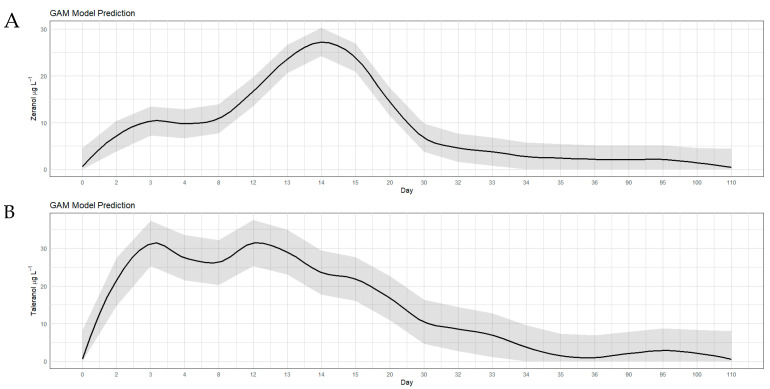
Concentrations of zeranol (**A**) and taleranol (**B**) in the urine of bovines that received zeranol implants used as a growth promoter at a concentration of 36,000 µg of zeranol for a period of 110 days (treatment B). The dark gray area surrounding the black line represents the confidence interval.

In the group of animals that received growth-promoting implants (treatment B), it was observed that zeranol levels increased rapidly from the second experimental day and reached a peak, with maximum concentrations of 27.24 µg L^−1^, from the thirteenth to fifteenth day (*p* < 0.05) (Figure 2; Table A2). After this period, a gradual decrease in zeranol levels was observed until the last experimental day (110th day), when it was still found in low concentration (0.40 µg L^−1^).

For taleranol, the same pattern was observed. However, the highest concentrations were observed from the fourth to the twentieth day, when they reached a peak of 31.40 µg L^−1^ (*p* < 0.05), and began to decrease, but were still quantified until the last experimental day (0.30 µg L^−1^). The levels of the metabolites zeranol and taleranol, which showed similar patterns throughout the study period, revealed that there is an inconsistent release of zeranol from the pellets and demonstrated the occurrence of oxidation of zeranol to zearalanone (ZAN) and subsequent reduction to taleranol. In this treatment, the presence of none of the three *Fusarium* spp. toxins (ZEN, α-ZEL and β-ZEL) was observed in the urine of the animals throughout the experimental period, demonstrating no contamination of the diet. In another study, Salvat et al. [10] analyzed the urine of animals that received zeranol implants and also observed a progressive increase in zeranol and taleranol from day one to day seven, with a peak of excretion on day ten; after that, a decrease in both metabolites was observed until the end of the experiment.

**Figure 3:**
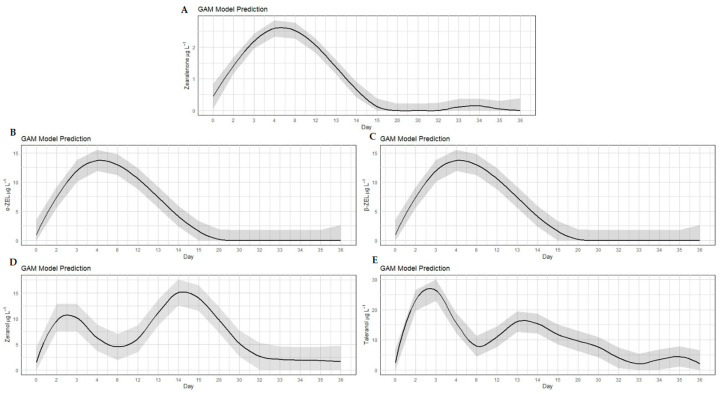
Concentrations of zearalenone (**A**), α-ZEL (**B**), β-ZEL (**C**), zeranol (**D**) and taleranol (**E**) in the urine of bovines that received zeranol implants and were fed with ZEN-contaminated corn at a concentration of 2100 ± 200 µg from the 1st to the 12th day of the experiment (treatment C). The dark gray area surrounding the black line represents the confidence interval.

Similar to treatment A, in the group of bovines that were implanted with zeranol and fed ZEN-contaminated corn from the first to the twelfth experimental day (treatment C), all analyzed RALs were detected. Peaks with the highest concentrations of ZEN, α-ZEL and β-ZEL were observed from the third to the twelfth day, with the highest average concentrations of 2.60 µg L^−1^, 1.92 µg L^−1^ and 13.70 µg L^−1^, respectively (*p* < 0.05) (Table A3). After that period, the levels of *Fusarium* spp. toxins in the urine of the animals decreased until they were no longer detected on day 20 of the experiment. For zeranol, two peaks (*p* < 0.05) were observed on days two and three (10.33 to 10.20 µg L^−1^, respectively) and from days 13 to 20 (11.16 to 9.75 µg L^−1^, respectively). For taleranol, the highest (*p* < 0.05) concentrations were observed on the second day (26.37 µg L^−1^), 3rd day (22.63 µg L^−1^) and fourteenth day (16.32 µg L^−1^) of the experiment. The excretion curves showed marked increases and decreases in the concentrations of the metabolites (Figure 3; Table A3).

### 2.2. Discrimination Between Abuse or Natural Contamination

**Figure 4:**
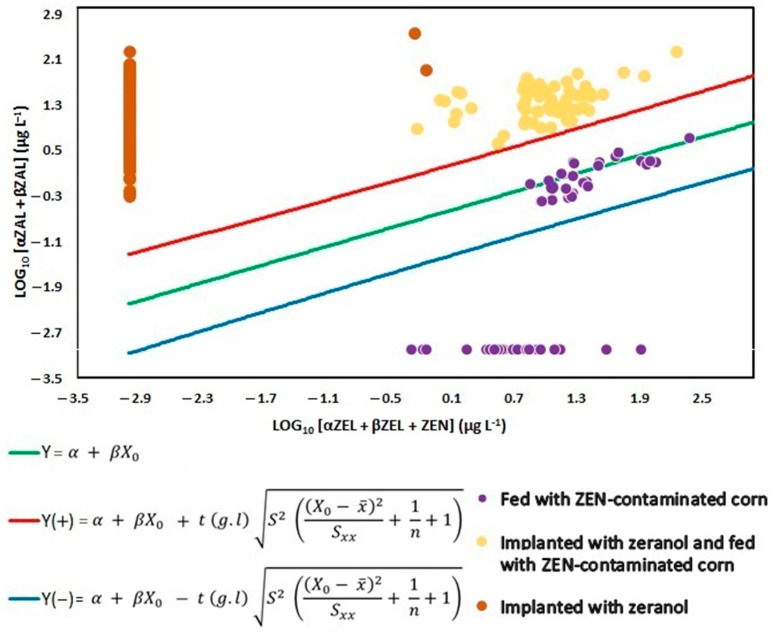
Distribution of metabolites of *Fusarium* spp. toxins in the urine of bovines that received zeranol implants, were fed contaminated corn, or both, according to the EURL equation considering a 99% confidence interval.

Discrimination between the illegal use of zeranol and the consumption of feed contaminated with *Fusarium* spp. toxin was based on comparing the sum of zeranol and taleranol concentrations with the sum of ZEN and α-ZEL and β-ZEL amounts. The quantitative outputs of the EURL equation (e.g., the calculated prediction intervals for each sample and where the results of the sum of zeranol/taleranol fell relative to that interval) are shown in Figure 4.

All the results of the zeranol implant (treatment B) and zeranol implant and corn contaminated with *Fusarium* spp. toxin (treatment C) fell within the 99% prediction interval (Figure 4) considering the EURL equation (above the red line). These results were expected because the animals received the hormone either by the implant or by feeding, even in lesser amounts in the latter (Table A1). The amount of metabolites detected in the group fed contaminated corn without implantation (treatment A) was significatively lower than in treatments B and C, which can be verified by the purple points located below the EURL equation (the red line), which is considered as the threshold. The aforementioned results demonstrate that the EURL equation was able to distinguish between the illegal use of zeranol and the consumption of food contaminated with *Fusarium* spp. toxin.

**Figure 5:**
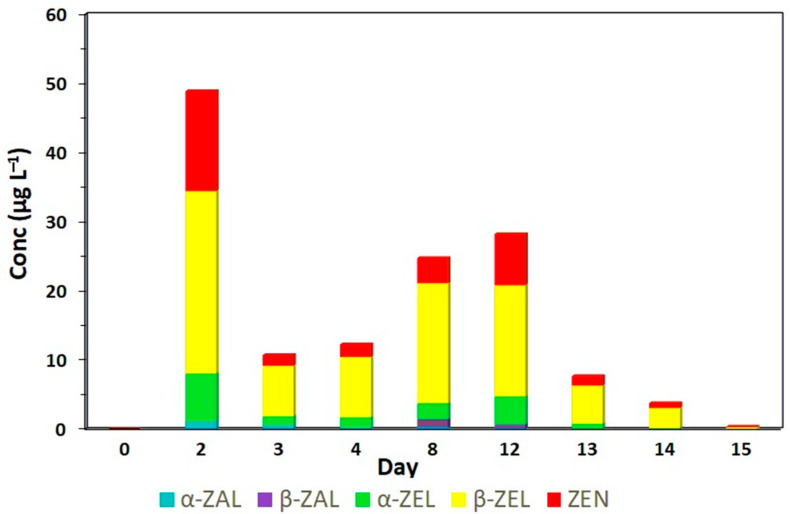
Profile of the five RALs: α-ZAL (zeranol), β-ZAL (taleranol), α- ZEL, β-ZAL and ZEN determined by LC-MS/MS analysis of the collected urine samples of bovines fed ZEN-contaminated corn at a concentration of 2100 ± 200 µg per day for 12 days (treatment A).

**Figure 6:**
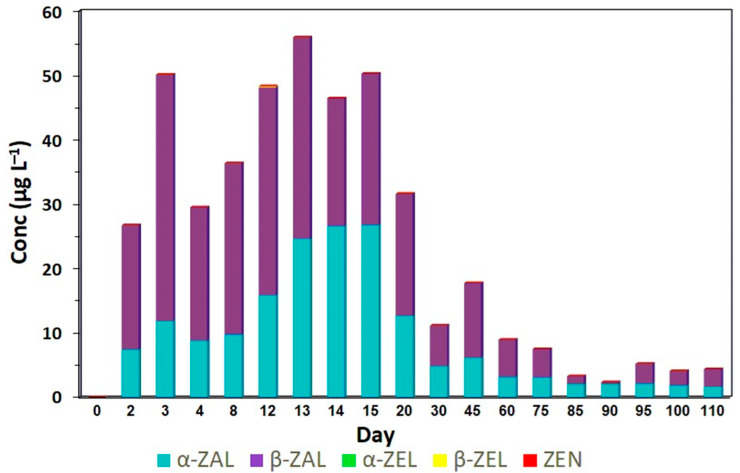
Profile of the five RALs: α-ZAL (zeranol), β-ZAL (taleranol), α-ZEL, β-ZAL and ZEN determined by LC-MS/MS analysis of the collected urine samples of bovines that received zeranol implants used as a growth promoter at a concentration of 36,000 µg of zeranol for a period of 110 days (treatment B).

**Figure 7:**
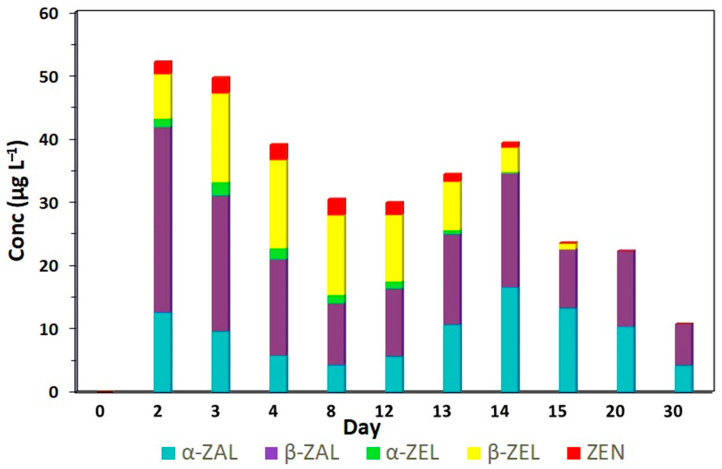
Profile of the five RALs: α-ZAL (zeranol), β-ZAL (taleranol), α-ZEL, β-ZAL and ZEN determined by LC-MS/MS analysis of the collected urine samples of bovines that received zeranol implants and were fed with ZEN-contaminated corn at a concentration of 2100 ± 200 µg from the 1st to the 12th day of the experiment (Treatment C).

None of the urine samples from animals fed only with *Fusarium*-contaminated corn (treatment A) was classified as indicative of zeranol abuse according to the EURL equation. From day two onward, the concentrations of ZEN, α-ZEL and β-ZEL consistently exceeded those of zeranol and taleranol (Figure 5). In the group of animals that received the zeranol implant (treatment B), the samples were classified as abusive use, from day two, after the placement of the implants until the last day evaluated, and the presence of *Fusarium* spp. toxins was not observed in any sample analyzed (Figure 6). According to Gençer et al. [22], zeranol has a longer withdrawal period and can be eliminated up to 65 days after implantation. The urine samples from animals that received the implant and were fed corn contaminated with *Fusarium* spp. toxins (treatment C) were also classified as abusive use of growth promoters. Although these samples presented high levels of *Fusarium* spp. toxins, the levels of zeranol and taleranol were higher and when the sum of zeranol and taleranol is greater than the sum of *Fusarium* spp. toxins, the samples are classified as abuse (Figure 7).

Although the results of this work are from an experiment with animals on a farm, the difference in the RAL profiles suggest the possibility of contamination with ZEN and/or the illegal administration of zeranol implants. The statistical model is a screening tool that can assist control laboratories and authorities in deciding if a non-compliant finding requires follow-up action or might be related to ingestion of *Fusarium* spp. toxins from contaminated feed. Consequently, the EURL mathematical model can be used to determine the illegal use of zeranol.

The differences in the quantifications of zeranol and taleranol versus ZEN plus α-ZEL and β-ZEL indicate natural contamination or abuse [23]. Teqja & Mavromati [24] used the EURL equation in urine samples from sheep, goat and swine to distinguish the illegal use of zeranol from the consumption of food contaminated with *Fusarium* spp. toxin zearalenone. They concluded that the feed was contaminated by ZEN and the model was appropriate to discriminate it from the illegal use of zeranol. Arrizabalaga-Larrañaga et al. [3] demonstrated that the EURL equation, already used to detect illegal zeranol in bovine urine, may also be used for porcine urine. This approach was tested in bile samples and yielded correct classifications [25].

## 3. Conclusions

The present study detected marked increases and decreases in the quantification of RALs in bovine urine over time. The metabolites simulated conditions that may arise in a production environment and demonstrated that the equation recommended by the EURL enabled differentiation between zeranol abuse and the ingestion of naturally contaminated corn. It can be concluded, therefore, that the use of the EURL equation can help competent authorities to decide whether a non-compliant RAL result requires follow-up action or may be related to the ingestion of contaminated feed.

## 4. Materials and Methods

### 4.1. Experimental Animals

A total of 24 crossbred bovines, 12 females and 12 males, were used. They had the same origin and age (18 months), a similar body weight (mean 180 kg for females and 200 kg for males) and were raised under similar environmental conditions. The animals were housed in separate barns according to treatments and sex. Before the experiment began, animals were submitted to an adaptation period of 15 days.

Before being given to the animals, all foods (grass silage and corn) were analyzed to detect the presence of ZEN using a liquid chromatography-tandem mass spectrometry (LC-MS/MS) methodology validated by the Commission Decision 2023/2782/EU [26], and the results showed the absence of *Fusarium* spp. toxins (ZEN, α-ZEL and β-ZEL). All animals were fed with 0.5 kg of corn and from 15 to 20 kg of silage per day, with mineral salt given “ad libitum” without protein supplementation. 

A portion of the corn was intentionally contaminated with ZEN, homogenized and analyzed to confirm the final amount of toxin present. Results showed a concentration of 4200 ± 400 µg kg^−1^, so the animals were fed corn contaminated with 2100 ± 200 µg of ZEN per day.

### 4.2. Treatments

Each treatment included four males and four females distributed in the following groups: A, fed with ZEN-contaminated corn; B, implanted with zeranol; and C, implanted with zeranol and fed with ZEN-contaminated corn.

The implants (Ralgro, Merck, Rahway, NJ, USA) were placed between the skin and cartilage on the back side of the ear, below the midline of the ear at the beginning of the experimental period. Three 12,000 µg zeranol implants were administered to each animal. The zeranol concentration, as reported by the manufacturer, was verified using LC-MS/MS.

The experimental periods of each treatment were established according to the time of exposure to ZEN and/or the zeranol implant. Treatment A lasted from 1 to 15 days (12 days of feeding with contaminated corn, followed by 3 more days of evaluation of uncontaminated feeding); treatment B lasted from 1 to 110 days (90 days of implant release, according to the manufacturer, followed by another 20 days); and treatment C lasted from 1 to 30 days (12 days of feeding with contaminated corn followed by an additional 18 days of evaluation of uncontaminated feeding, but still with the implant).

On day zero (control), urine was collected from all animals before the treatments began. The animals received the implant and the contaminated corn feed on day one. Subsequently, collections were made on days 2, 3, 4, 8, 12, 13, 14, 15, 20, 30, 45, 60, 75, 85, 90, 95, 100 and 110. The urine samples were individually collected, stored at −20 °C and sent to the Federal Laboratory of Animal and Plant Health and Inspection in São Paulo (LFDA-SP) where all the urine samples were analyzed.

This study was carried out in strict accordance with the recommendations of the National Council for the Control of Animal Experimentation (CONCEA) at the Brazilian Ministry of Science and Technology and Innovation (MCTI). The protocol was approved by the Ethics Committee in Animal Experimentation at the Federal University of Minas Gerais State (UFMG) (Protocol Number: 258/2018).

### 4.3. Detection and Quantification of RALs

#### 4.3.1. Laboratory Analyses

The presence and levels of zeranol, taleranol, ZEN and its metabolites α-ZEL, β-ZEL, in urine were assessed using a QuEChERS extraction method and instrumental analysis with LC-MS/MS.

#### 4.3.2. Chemicals and Reagents

Analytical standard (Certified Reference Material—CRM) zearalenone, α-ZEL, β-ZEL, zeranol, and taleranol were obtained from National Measurement Institute (Canberra, Australia), whereas Estradiol-d4 (internal standard) was purchased from Toronto Research Chemicals (Toronto, Canada).

Acetonitrile (HPLC grade, JT Baker, Center Valley, PA, USA), methanol (HPLC grade), magnesium sulfate anhydrous (Scharlau, Barcelona, Spain), acetic acid (CH_3_COOH, Emsure Grade), formic acid (Merck, Darmstadt, Germany), sodium acetate anhydrous (Neon, Sao Paulo, Brazil), ammonium acetate (NH_4_CH_3_CO_2_), β-Glucuronidase (type HP-2 from *Helix Pomatia*) and primary secondary amine (PSA) (Sigma-Aldrich, St. Louis, MO, USA) were used.

#### 4.3.3. Instrumentation

The analyses were performed using a Waters Technologies ACQUITY UPLC I-Class system coupled to an Xevo TQ-XS Triple Quadrupole mass spectrometer (Waters, Milford, MA, USA). An InfinityLab Poroshell 120 EC-C18 (50 × 3.0 mm, 2.7 μm) was used for chromatographic separation at 40 °C. Mobile phase A consisted of water, and mobile phase B used methanol at a flow rate of 0.4 mL min^−1^. Initial conditions were set at 50% A and B with a linear gradient from 50% A to 40% A for 3 min and from 40% A to 35% for 2 min and returned to 50% A from 5.0 min to 5.6 min. Then, 50% A was maintained until 7.0 min. The injection volume was 10 μL.

The operational conditions of the mass spectrometer were established by direct infusion of the standards. Two product ions, one for quantitation and the other for confirmation, were established and monitored for each analyte. The analyses were performed using electrospray ionization (ESI) in the negative ion mode. Nitrogen was used as the desolvation and cone gas with flowrates of 1000 L h^−1^ and 150 L h^−1^, respectively. The desolvation temperature was maintained at 500 °C, the capillary voltage was set at −2.5 kV and the nebulizer gas flow was set at 7 bars. Argon was used as collision gas, and the flow rate was set at 0.15 mL min^−1^.

#### 4.3.4. Samples and Extraction Procedure

Sample preparation and cleanup were performed using a modified QuEChERS method [27]. The samples were homogenized, and a 5.0 mL aliquot was pipetted into a 50 mL polypropylene centrifuge tube; 5.0 mL of acetate buffer 1 mol/L and 10 µL of β-Glucuronidase were added. After homogenization, the samples were incubated at 37 °C for nearly 16 h (overnight).

The tubes were removed from the incubator and allowed to cool to room temperature, then 8 mL of acetonitrile and 3 g of MgSO_4_:NaCH_3_COO (2:1) were added. The tubes were vortexed for 30 sec and then centrifuged (4000 rpm) for 10 min.

A volume of 6 mL of organic extract was transferred to a 15 mL polypropylene centrifuge tube containing 1 g of MgSO_4_:PSA (3:1), vortexed for 30 sec and centrifugated at 4000 rpm for 10 min. A volume of 4 mL of liquid phase was transferred to a glass tube and evaporated to dryness at 40 °C under nitrogen (N_2_). The dry extracts were reconstituted in 1 mL of MeOH:H_2_O (50:50, *v/v*), immersed in an ultrasonic bath for 5 min, and vortexed for 30 s. Afterwards, the samples were filtered through a filter unit with a PTFE membrane (pore size of 0.22 μm, Saint Vallen, Brasilia, Brazil), and then, 100 µL of the filtered solution were diluted in 400 µL of ultrapure water and reserved for injection.

### 4.4. Discrimination Abuse or Contamination Through the Statistical Model

The equation proposed by the EURL that distinguishes abusive use of the growth promoter zeranol from natural contamination with *Fusarium* spp. toxins was used to interpret the results obtained. This model relates the sum of the concentrations of zeranol and taleranol (response variable) with the concentrations of *Fusarium* spp. toxins (explanatory variable) transformed into logarithms to base 10 [18]. To calculate the prediction interval, the following equation was used:

(1) $$\alpha +\beta x_{0}\;\pm \;t\;(d.f.)\sqrt{s^{2}\left(\frac{{{(x}_{0}-\bar{x})}^{2}}{S_{xx}}+\frac{1}{n}+1\right)}$$

where *n* is the number of observations and *t* (*d.f.*) is the desired percentage point of the *t* distribution. Thus, for a prediction interval of, for example, 99% and an *x* value of *x*_0_, the above equation will give two values between which we are 99% confident that the true value should lie for this *x* value.

A linear regression was conducted using the logarithmic (base 10) transformation of both the sum of zeranol and taleranol concentrations and the sum of ZEN, α-ZEL, and β-ZEL (*Fusarium* spp. toxin concentration) [18]. The regression parameters and their associated standard errors allowed the calculation of a 99% prediction interval for the sum of zeranol and taleranol concentrations, given any value of the sum of ZEN, α-ZEL, and β-ZEL, following the method described by McConway et al. [28]. The equation derived by the EURL considered −0.6000 as the intercept (*α*) and 0.5318 as the slope (*β*).

### 4.5. Statistical Analyses

The experimental design was completely randomized in split plots, with the plot consisting of the treatments and the subplots consisting of the days of urine collection. Statistical analysis was performed using R language [29]. As no differences were observed between the sexes, each treatment had eight replicates of one bovine each. The means were compared using the Tukey test at 5% probability.

Generalized Additive Models (GAM) were adjusted due to the nonlinear nature of the results of each treatment over the days of urine collection; no models comparing treatments were created. GAM are an extension of Generalized Linear Models (GLM), designed to capture nonlinear relationships between predictor variables and the response variable. In this model, the relationship between the independent and dependent variables is expressed as a sum of smooth functions (splines), providing greater flexibility in modelling, without the need to assume a specific shape for the curve. The Gaussian distribution family was used due to the continuous nature of the metabolites, which are the dependent variable. One of the limitations of models based on normal distribution and continuous data is the possibility of negative values occurring, something which is not possible in the context of this study. Thus, once the model’s fit was verified, the graphs presented were fixed at values greater than or equal to zero. The collection days, which are the independent variables, were entered into the model as a discrete variable relating to the collection order, such as first collection, second collection, and so on [30].

To deal with repeated measurements on the same animals at different times, random effects models were fitted. An animal identification (ID) variable was created and incorporated as a random effect, allowing the capture of individual variations between animals.

The smoothing terms of the model (Base Dimension-*k*) were evaluated through the *p*-values calculated based on the effective degrees of freedom (edf), indicating the significant contribution of the smooth function to the model when *p* < 0.05. The fit of the model and its residuals were analyzed using the gam.check() function, which evaluates residual patterns and the *k*-index to verify the adequacy of the smoothing terms. Models with a *k*-index close to 1 and *p*-values greater than 0.05 indicate a balanced fit in terms of smoothness and complexity. Multiple comparisons within the treatments across different collection orders were performed using the emmeans package, with Sidak adjustment and a 95% confidence level.

For ZEN, in treatment A, the smoothing terms of the GAM model did not show statistical significance and were then analyzed using a linear regression model.

## Data Availability

The original contributions presented in this study are included in the article. Further inquiries can be directed to the corresponding authors.

## Appendix A

**Table A1:** Concentrations of ZEN, α-ZEL, β-ZEL, zeranol and taleranol residues in the urine of bovines fed ZEN-contaminated corn at a concentration of 2000 ± 200 µg per day for 12 days.

Resorcylic Acid Lactones					
Day	Zearalenone	α-Zearalenol	β-Zearalenol	Zeranol	Taleranol
0	ND	ND	ND	ND	ND
2	14.45 ^a^	6.24 ^a^	24.37 ^a^	1.08 ^a^	ND
3	1.54 ^b^	1.92 ^b^	9.10 ^bcd^	0.65 ^ab^	ND
4	1.81 ^b^	1.28 ^b^	8.63 ^bcd^	0.26 ^bc^	ND
8	3.63 ^ab^	2.49 ^ab^	17.14 ^ab^	0.32 ^bc^	1.01 ^a^
12	7.40 ^ab^	3.74 ^ab^	15.80 ^abc^	0.19 ^bc^	0.52 ^b^
13	1.34 ^b^	1.02 ^b^	6.04 ^bcd^	ND	ND
14	0.68 ^b^	0.13 ^b^	2.71 ^cd^	ND	ND
15	0.07 ^b^	ND	0.34 ^d^	ND	ND

Means follow by distinct letters in the same columns are different according to the Tukey test (*p* < 0.05). ND: not detected.

**Table A2:** Concentrations of ZEN, α-ZEL, β-ZEL, zeranol and taleranol in the urine of bovines that received zeranol implants used as a growth promoter at a concentration of 36,000 µg of zeranol for a period of 210 days.

Resorcylic Acid Lactones					
Day	Zearalenone	α-Zearalenol	β-Zearalenol	Zeranol	Taleranol
0	ND	ND	ND	ND	ND
2	ND	ND	ND	7.13 ^defgh^	21.35 ^abc^
3	ND	ND	ND	10.35 ^cdef^	31.40 ^a^
4	ND	ND	ND	9.80 ^cdefg^	27.45 ^a^
8	ND	ND	ND	10.86 ^cde^	26.26 ^a^
12	ND	ND	ND	16.66 ^bc^	31.40 ^a^
13	ND	ND	ND	23.60 ^ab^	29.00 ^a^
14	ND	ND	ND	27.24 ^a^	23.56 ^ab^
15	ND	ND	ND	23.90 ^ab^	21.83 ^abc^
20	ND	ND	ND	14.43 ^cd^	16.78 ^abcd^
30	ND	ND	ND	6.80 ^efgh^	10.50 ^bcde^
45	ND	ND	ND	4.61 ^efgh^	8.56 ^bcde^
60	ND	ND	ND	3.77 ^efgh^	6.97 ^cde^
75	ND	ND	ND	2.74 ^fgh^	3.73 ^de^
85	ND	ND	ND	2.43 ^gh^	1.45 ^de^
90	ND	ND	ND	2.15 ^gh^	1.04 ^de^
95	ND	ND	ND	2.08 ^gh^	2.02 ^de^
100	ND	ND	ND	2.11 ^gh^	2.99 ^de^
110	ND	ND	ND	1.42 ^h^	2.39 ^de^

Means follow by distinct letters in the same columns are different according to the Tukey test (*p* < 0.05). ND: not detected.

**Table A3:** Concentrations of ZEN, α-ZEL, β-ZEL, zeranol and taleranol in the urine of bovines that received zeranol implants and were fed with contaminated ZEN from the 1st to the 12th day of the experiment.

Resorcylic Acid Lactones					
Day	Zearalenone	α-Zearalenol	β-Zearalenol	Zeranol	Taleranol
0	ND	ND	ND	ND	ND
2	1.60 ^cde^	1.38 ^bc^	7.32 ^def^	10.33 ^abc^	26.37 ^a^
3	2.40 ^ab^	1.92 ^ab^	11.88 ^abc^	10.20 ^abc^	22.63 ^ab^
4	2.60 ^ab^	1.92 ^ab^	13.70 ^ab^	6.26 ^bcd^	14.60 ^bc^
8	2.40 ^ab^	1.60 ^ab^	12.93 ^ab^	4.52 ^cd^	9.60 ^cd^
12	1.93 ^bcd^	1.20 ^abcd^	10.56 ^bcd^	6.09 ^bcd^	10.26 ^cd^
13	1.30 ^e^	0.80 ^cde^	7.40 ^cde^	11.16 ^ab^	14.23 ^bc^
14	0.64 ^f^	0.42 ^e^	4.17 ^fg^	15.07 ^a^	16.32 ^abc^
15	0.21 ^f^	ND	1.60 ^h^	13.99 ^a^	10.40 ^cd^
20	ND	ND	ND	9.75 ^abc^	10.49 ^cd^
30	ND	ND	ND	5.19 ^bcd^	6.50 ^cd^

Means follow by distinct letters in the same columns are different according to the Tukey test (*p* < 0.05). ND: not detected
